# Efficacy of auricular point-vagus nerve stimulation in adolescent insomnia patients and the brain function regulation mechanisms: a study protocol for an experimental, randomized, controlled clinical trial

**DOI:** 10.3389/fneur.2025.1643509

**Published:** 2025-09-17

**Authors:** Wei He, Lu Guan, Yuhong Jiang, Zi Guo, Xiaomei Shao, Jianqiao Fang, Junfan Fang, Junying Du

**Affiliations:** Department of Neurobiology and Acupuncture Research, The Third School of Clinical Medicine, Zhejiang Chinese Medical University, Hangzhou, China

**Keywords:** acupuncture, auricular point, vagus nerve, adolescent insomnia, randomized controlled trial, protocol

## Abstract

**Background:**

Adolescent insomnia is a prevalent issue with significant implications for mental and physical well-being. Given the increasing incidence of sleep disorders, there is an urgent need for safe and efficient treatment modalities, particularly non-pharmacological interventions. Auricular point therapy, a prominent element of acupuncture in China, is often employed to address insomnia. However, there remains a lack of research on the efficacy of auricular point-vagus nerve stimulation in managing insomnia among teenagers. To evaluate the efficacy of this interventions and explore the regulatory mechanisms of brain function, a randomized trial is planned.

**Methods/design:**

This trial is a single-center, single-blind, randomized controlled study. A total of 174 adolescent patients with insomnia will be randomly assigned to either the treatment or control groups. Over a period of 4 weeks, patients in the experimental group will undergo bilateral auricular point-vagus nerve stimulation, while those in the control group will receive bilateral non-auricular acupoint-vagus nerve stimulation. Data collection will occur at baseline, 1 week into the intervention, 4 weeks post-treatment initiation, and 4 weeks post-treatment completion. The primary outcome measures will include the Pittsburgh Sleep Quality Index (PSQI), Adolescent Sleep Hygiene Habits Scale (ASHS), and Adolescent Sleep Assessment Questionnaire (DSM). Secondary outcome measures encompass the Self-Assessment Scale for Anxiety (SAS), Self-Depression Scale (SDS), Quality of Life Scale (SF-36), and Autonomic Composite Score (COMPASS-31). Other indicators will involve the use of functional magnetic resonance imaging (fMRI), heart rate variability (HRV), and polysomnography (PSG).

**Discussion:**

The findings of this study will provide strong support for the use of auricular-vagus nerve stimulation as a safe and effective non-pharmacological intervention for insomnia. This approach will offer advantages over medication by mitigating the risks of addiction and adverse effects associated with prolonged drug therapy. Furthermore, the results will align with prior research that underscores the positive impact of auricular acupuncture point stimulation on enhancing sleep quality.

**Clinical trial registration:**

https://www.chictr.org.cn, ChiCTR2400087889.

## 1 Introduction

Sleep is a critical physiological necessity for humans and plays a pivotal role in both physical and mental well-being. The increasing prevalence of sleep disturbances, notably insomnia, among adolescents in recent years significantly affects teenagers’ educational attainment and quality of life (1). Studies have reported varying rates of insomnia among Chinese adolescents, ranging from 19.3 to 38.2%, showing a gradual increase over time (2, 3). A meta-analysis of sleep patterns worldwide among 11–18-year-olds revealed that adolescents frequently experience sleep quality problems such insomnia and trouble falling asleep in addition to sleep deprivation (4). Beyond its impact on daytime functioning, insomnia is closely associated with psychological challenges such as depression and self-harming tendencies (5, 6). Hence, there is a pressing need in public health to explore effective interventions for adolescent insomnia.

Based on their pharmacodynamic effects, the medications currently employed for treating insomnia are primarily categorized into four groups: benzodiazepine receptor agonists, melatonin receptor agonists, histamine receptor antagonists, and orexin receptor antagonists (7). Despite their rapid onset of action, prolonged utilization can result in addiction, drug resistance, and various adverse health effects, particularly in teenagers (8). Non-pharmacological interventions represent a substantial progression in the quest for treatment that are less detrimental and more acceptable to this age group.

Excessive autonomic nervous system activity, elevated neurogenic inflammatory factors, and heightened sensitivity to various stimuli are key contributors to insomnia (9, 10). Strong correlations exist between vagus nerve activity and sleep quality (11). Vagus nerve stimulation can directly modulate autonomic nervous system balance, activate the body’s cholinergic neuron system, and reduce inflammatory mediator release (12), making it an effective insomnia treatment. However, surgical risks, postoperative complications, and high device revision rates have limited the widespread adoption of implantable vagus nerve stimulation (13). Auricular point-vagus nerve stimulation offers a non-invasive alternative, circumventing these concerns. The auricular branch of the vagus nerve is distributed across the inner half of the ear, the only superficial vagal projection area on the body (14). Key acupoints in this region, such as *Heart*, *Spleen*, *Liver*, *Kidney*, *Shenmen*, and *Sympathetic* are frequently used in insomnia treatment. The external auricular concha, with its thin and highly sensitive skin, is the optimal surface site for non-invasive vagus nerve stimulation and has shown notable clinical efficacy in adult patients (15).

While some studies suggest that auricular vagus nerve stimulation may increase cortical arousal and alertness, potentially counteracting its benefits for insomnia (16). Others indicate that these effects depend on stimulation intensity and frequency. Low-intensity, low-frequency stimulation produces only transient arousal, whereas repeated vagus nerve stimulation enhances parasympathetic tone, ultimately promoting sleep (17, 18). Given the complexity of auricular vagus nerve stimulation and the lack of clarity regarding its efficacy in adolescents with insomnia, this study aimed to investigate the neurological processes involved in auricular vagus nerve stimulation and assess its therapeutic potential for teenage insomnia.

## 2 Methods and analysis

### 2.1 Study design

**Figure 1:**
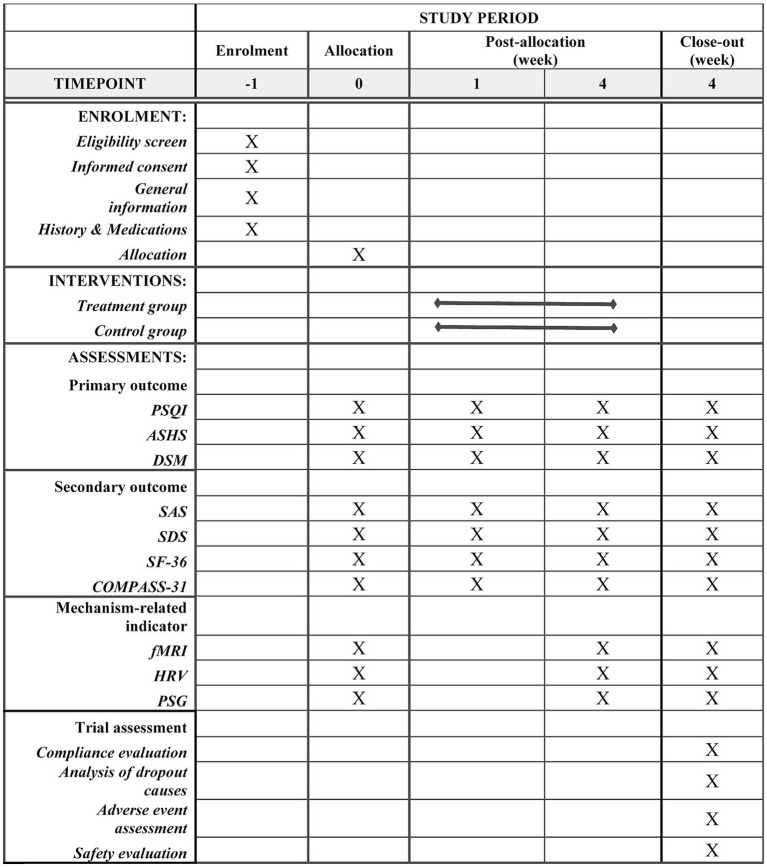
SPIRIT schedule.

**Figure 2:**
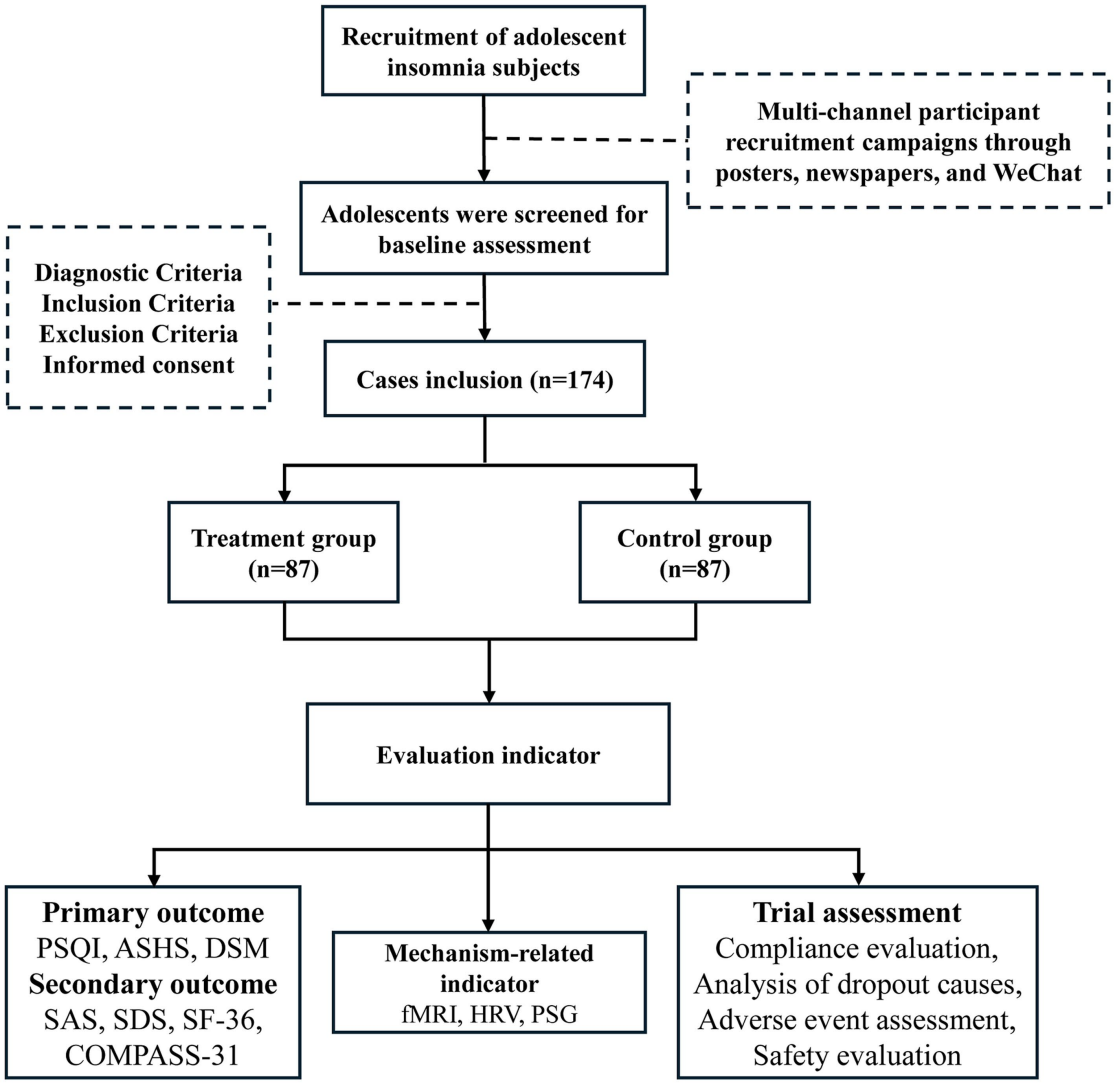
Trial flow chart.

On August 6, 2024, the study was registered in the Chinese Clinical Trial Registry.^1^ This research is a randomized controlled prospective single-blind clinical trial. A total of 174 participants were sought in Zhejiang Province. The dynamic randomization method of a central randomization system was used to randomly group the subjects who met the inclusion criteria. The placebo group is selected as the control group. Electroacupuncture will be used to stimulate bilateral auricular point-vagus nerve locations in the treatment group and bilateral non-auricular-vagus distribution areas in the control group. The evaluation of therapeutic indicators is carried out by personnel who are unaware of the grouping situation, and the separation of researchers, operators, and statisticians is implemented. The 8-week trial is divided into 4 weeks of therapy and 4 weeks of follow-up. The Chinese Guidelines for the Diagnosis and Treatment of Insomnia Disorders, which were compiled by the Chinese Sleep Research Society in 2019, provided the relevant criteria used to develop the study protocol (No.1.0, Date: 2024.01.15). Further details are provided in Figures 1, 2.

### 2.2 Study setting

The study is conducted at Third Affiliated Hospital of Zhejiang Chinese Medical University, which leads the group and provides the medical resources and expert staff required to support the project’s execution. The Department of Acupuncture and Moxibustion, which has skilled medical personnel and cutting-edge therapeutic equipment suitable for undertaking the study of auricular-vagus nerve stimulation therapy, hosts the investigation. The intervention is administered to participants in a hospital treatment room that has been reserved. To ensure the safety and effectiveness of the therapy, the treatment room is equipped with the necessary electroacupuncture and monitoring equipment. The hospital creates a calm, cozy environment that encourages participant relaxation and minimizes the impact of external factors on the treatment’s efficacy. The humidity, light, and temperature of the treatment room is controlled to the appropriate degree.

### 2.3 Ethics approval

This trial protocol had been approved by the Medical Ethics Committee of the Third Affiliated Hospital of Zhejiang University of Traditional Chinese Medicine (ZSLL-KY-2024-018-01), and registered in https://www.chictr.org.cn (Identifier: ChiCTR2400087889). This research complies with the Helsinki Declaration. Prior to the start of the study, hospital authorization had been secured. The ethics committee supervises and evaluates the design and execution of the trial procedures.

### 2.4 Sample size

Non-inferiority sample size estimation for two independent samples was conducted via the Powerandsamplesize.com network. Using the PSQI score as the primary outcome, the sample size was calculated to require a minimum of 78 individuals in each group. Considering the 10% dropout rate in outpatient clinics, the sample size for each group would need to be expanded to 87, totaling 174 people. The specific calculation formula is as follows:

$$n_{A}={\mathrm{\kappa n}}_{B},n_{B}=(1+\frac{1}{\kappa }){\left(\sigma \frac{z_{1-\alpha }+z_{1-\beta }}{\mu_{A}-\mu_{B}-\delta }\right)}^{2}$$

$$1-\beta =\Phi (z-z_{1-\alpha })+\Phi (-z-z_{1-\alpha }),z=\frac{\mu_{A}-\mu_{B}-\delta }{\sigma \sqrt{\frac{1}{n_{A}}+\frac{1}{n_{B}}}}$$

### 2.5 Participant recruitment

The recruitment period for participants ran from September 1, 2024, to March 31, 2025, following registration and ethical approval. A formal informed consent form must be signed for eligible participants to take part and the written consent of their legal guardian must be obtained.

### 2.6 Diagnostic criteria

This study adopted the relevant standards stipulated in the “Guidelines for the Diagnosis and Treatment of Insomnia Disorders in China” compiled by the Chinese Sleep Research Society in 2019. Diagnostic criteria: (a) Chief complaints: inability to fall asleep, persistent inability to fall asleep, waking up earlier than intended, refusal to go to bed at the appointed hour, and inability to go asleep on one’s own without assistance from a parent or caregiver. (b) Deficits in daytime functioning: weariness or low energy; difficulty focusing, paying attention, or remembering things; impairment in social, familial, professional, or academic functioning; agitation or irritability of mood; daytime sleepiness; behavioral issues (such as hyperactivity, impulsivity, or aggression); lack of drive, energy, or motivation; vulnerability to errors or mishaps; and anxiety regarding the quality of sleep. (c) Inappropriate sleep opportunities (e.g., adequate sleep periods) or sleep surroundings (e.g., dark, quiet, safe, pleasant places) cannot account for all of these irregularities in sleep and wakefulness. (d) Chronic insomnia: >3 episodes per week for at least 3 months; unrelated to other sleep problems.

### 2.7 Inclusion criteria

Patients were included if they simultaneously met all seven of the criteria: (1) Individuals who meet the diagnostic standards listed above for insomnia. (2) Age: 13–17 years old; no restrictions on gender (2). (3) A score of at least seven on the Pittsburgh Sleep Quality Index Rating Scale (PSQI). (4) Signed informed consent was obtained and obtained the written consent of their legal guardian. (5) Individuals who are able to strictly comply with the treatment requirements. (6) No major illnesses affecting the blood system, immunity, or internal organs illnesses, without any mental health issues. (7) Within 1–3 weeks, no related drugs such as benzodiazepines and melatonin were taken (based on the elimination half-life).

### 2.8 Exclusion criteria

The exclusion criteria are as follows: (1) Patients who were inadvertently registered despite not meeting the eligibility requirements. (2) Patients who are under 13 years old or over 17 years old. (3) Individuals with a history of head trauma, neurological conditions, and other objective environmental causes of insomnia. (4) Individuals with severe underlying illnesses, such as endocrine, hematologic, or digestive system cancers, and patients whose conditions vagal nerve stimulation, such as hyperthyroidism and heart failure. (5) Patients with a PSQI score <7. (6) History of drug abuse or alcoholism. (7) Skin allergy, injury or inflammation at the site of auricular application. Women who are pregnant or preparing for pregnancy, or women who are breastfeeding. (8) Participation in other clinical trials in the last 6 months, or patients who have taken anticholinergic drugs or hormonal drugs in the recent past, or patients who are taking drugs to inhibit the cerebral cortex or the nervous system on an irregular basis.

### 2.9 Intervention and comparison

#### 2.9.1 Treatment group

**Figure 3:**
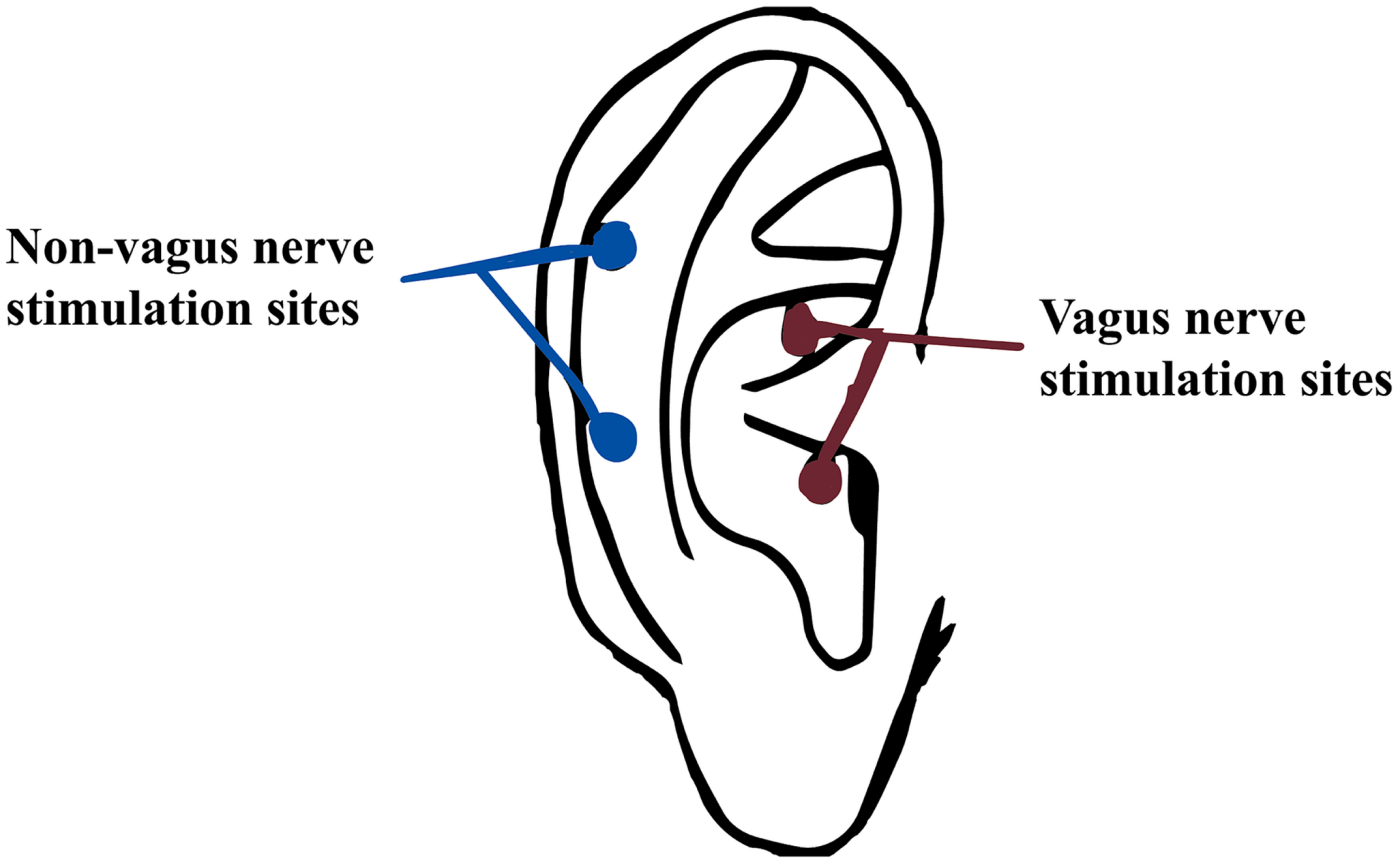
Non-vagus nerve stimulate sites and vagus nerve stimulation sites.

**Figure 4:**
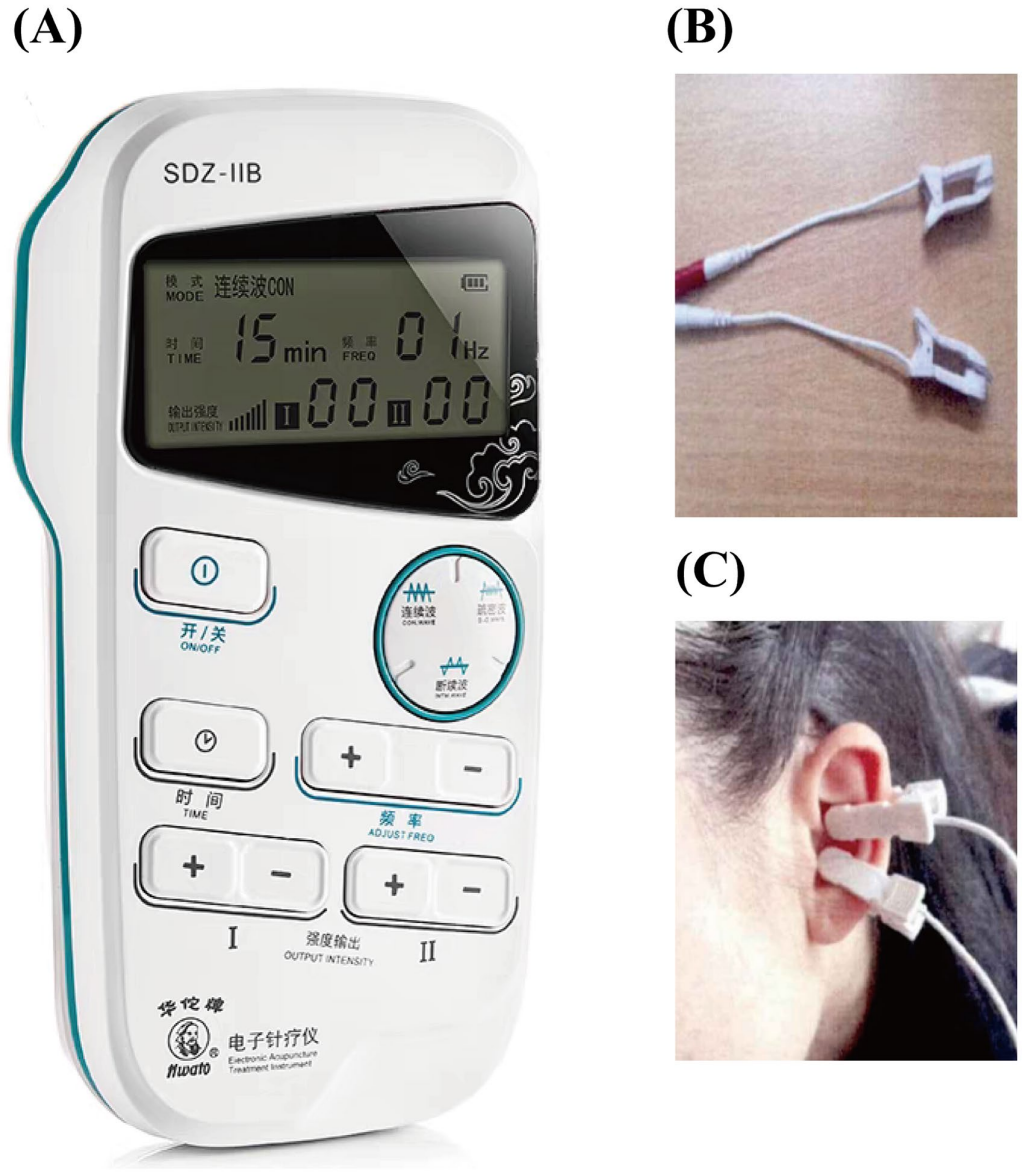
Treatment diagram. **(A)** Treatment apparatus; **(B)** ear clip; **(C)** treatment location.

The SDZ-IIB electronic acupuncture instrument (Hwato brand, Suzhou Medical Appliance Factory, China) is used for auricular point-vagus nerve stimulation. The stimulation site is the vagus nerve distribution area on both sides of the body’s ear acupuncture points, as detailed in Figures 3, 4. The treatment parameters are as follows: sparse and dense wave, 4 Hz/20 Hz, biphasic pulse with a 0.2 ms pulse width, stimulation intensity according to the patient’s tolerance level (characterized by mild tingling without discomfort). Treatment will be administered daily at noon and before bedtime for 1 h, with each treatment lasting 30 min. Treatment will be provided 5 days a week for four consecutive weeks. Patients will be trained by a medical expert to ensure that each patient or a member of their family can correctly use the device on their own and then take it home to receive treatment. Observation time points are pre-treatment baseline, 1 week into treatment, 4 weeks into treatment, and 4 weeks following the conclusion of treatment.

#### 2.9.2 Control group

The stimulation site is the bilateral non-auricular vagus nerve distribution area (Figure 3). The instrument, treatment process and treatment parameters are the same as those for the treatment group. Observation time points are pre-treatment baseline, 1 week into treatment, 4 weeks into treatment, and 4 weeks following the conclusion of treatment (follow-up).

### 2.10 Hypotheses and outcomes

#### 2.10.1 Hypotheses

After 4 weeks of treatment, the adolescents in the treatment group showed significant improvement in sleep quality compared with the control group. Including the total sleep time will increase, the time required to fall asleep will decrease, the frequency of easy awakening at night and early awakening will decrease, and the conditions of feeling sleepy, lacking energy and having difficulty concentrating in daily life will be improved and so on.

#### 2.10.2 Primary outcome

In this study, the Pittsburgh Sleep Quality Index Score (PSQI), the Adolescent Sleep Hygiene Scale (ASHS), and the Adolescent Sleep Assessment Questionnaire (DSM) are used to comprehensively evaluate treatment-related changes in sleep quality. PSQI: A global measure assessing overall sleep quality through subjective sleep parameters and disturbances. ASHS: Specifically evaluates behavioral and environmental factors influencing adolescent sleep patterns. DSM: Focuses on clinical characterization of sleep disorders, including typology and severity.

##### 2.10.2.1 PSQI

The PSQI includes seven sections with scores from 0 to 3, yielding a total score ranging from 0 to 21, where a higher score indicates poorer sleep quality (19).

##### 2.10.2.2 ASHS

The ASHS contains nine sections with scores from 1 to 6, a higher score indicates better sleep hygiene (20).

##### 2.10.2.3 DSM

The questionnaire is based on the *Diagnostic and Statistical Manual of Mental Disorders*. It can help assist in identifying sleep disorder, assess the specific manifestations and impacts of sleep problems and determine whether the treatment is effective and whether it meets the clinical criteria for symptom relief (21).

#### 2.10.3 Secondary outcome

##### 2.10.3.1 Self-rating anxiety scale

The self-rating anxiety scale (SAS) contains 20 items with scores from 1 to 4. The total score is obtained by adding up the scores of each item and then multiplying the sum by 1.25. The higher the score, the more severe the patients’ anxiety symptoms (22).

##### 2.10.3.2 Self-rating depression scale

The self-rating depression scale (SDS) contains 20 items with scores from 1 to 4. The SDS index is calculated by adding up the scores of each item and then dividing the total by 80. The higher the SDS index, the more severe the patients’ depression (23).

##### 2.10.3.3 Quality of life scale

The quality of life scale (SF-36) can compare the changes in patients’ physical and psychological health before and after treatment (24).

##### 2.10.3.4 Composite autonomic score

The composite autonomic score (COMPASS-31) can compare the difference in patients’ autonomic nerve before and after treatment. The higher the score, the more severe the autonomic nerve symptoms (25).

#### 2.10.4 Mechanism-related indicator

##### 2.10.4.1 Functional magnetic resonance imaging

The functional magnetic resonance imaging (fMRI) can reflect the neural activity indirectly by detecting the changes in blood flow and oxygenation levels during brain activity. GE Discovery 3.0 T MR750w scanner (General Electric, America) with a 20-channel head coil will be used. An echo-planar imaging sequence will be used to acquire the resting-state functional data with the following parameters: TR/TE = 2,000/40 ms, flip angle = 90°, voxel size = 3.75 × 3.75 × 4.00 mm^3^, matrix = 64 × 64, slice thickness = 3.0 mm, number of slices = 40. In addition, a three-dimensional turbo fast echo T_1_WI sequence will be used to get the structural images, following are the parameters: TR/TE = 8.0/3.1 ms, matrix = 256 × 256, slice thickness = 1.0 mm.

To study the regulatory mechanism of auricular point-vagus nerve stimulation on the brain function of adolescent patients with insomnia, the analysis will be conducted from three aspects: amplitude of low frequency fluctuation (ALFF), regional homogeneity (ReHo) and functional connectivity (FC). Twenty subjects will be randomly selected from each group. The observation time points are before the treatment baseline, 4 weeks after the treatment, and 4 weeks after the treatment ended. We refer to the method proposed by Zhang et al. to calculate ALFF, ReHo and FC (26). Before calculating ALFF, ReHo and FC, the original images will undergo preprocessing. Preprocessing is based on the MATLAB R2013b platform using RESTplus1.2 software (REST^2^). To ensure the accuracy of the data, the first 10 images will be removed. Then, time correction and head movement correction will be carried out. After that, align functional images with the corresponding structural images. They will be spatially normalized to the Montreal Neurological Institute (MNI) template to locate the active areas of the brain. Finally, the acquired signal will undergo low-pass filtering within the range of 0.01–0.08 Hz to eliminate interference caused by high-frequency and low-frequency signals. To calculate ALFF, the functional image would first be converted into the frequency domain by using the Fast Fourier Transform to obtain the power spectrum. The square root of the power spectrum will be averaged across 0.01–0.08 Hz at each voxel and each voxel’s ALFF will be divided by the global mean of the ALFF to obtain the standardized ALFF. Unsmoothed data will be used to calculate ReHo through Kendall consistency coefficients to measure the synchronization of the time series of a voxel with its 26 nearest neighboring voxels. Each ReHo map will be divided by the global mean ReHo for standardization and will be smoothed using a Gaussian kernel with a 6 mm FWHM. The FC value is calculated as the linear correlation coefficient (Pearson Correlation) of the BOLD signal time series of two voxels. It will be computed by the RESTplus.

##### 2.10.4.2 Heart rate variability

The heart rate variability (HRV) can assess autonomic nerve function and stress recovery ability. It can help to analyze the patients’ depression, anxiety and fatigue conditions. If patients’ HRV become higher after the treatment, it can prove that the patients have received better rest and their mental conditions have become more stable. We will obtain precise heartbeats’ timing points through electrocardiogram, and calculate the root mean square of the differences between adjacent RR intervals to obtain the value of Root Mean Square of Successive Differences (RMSSD). RMSSD is a key HRV indicator. The higher the RMSSD, the stronger the vagus nerve tension and the better the stress adaptation ability.

##### 2.10.4.3 Polysomnography

Including electroencephalo-graph (EEG, distinguishing sleep stages and monitoring abnormal electroencephalogram activity), electro-oculogram (EOG, detecting REM sleep and eye movement), electromyography (EMG, monitoring the muscle tension of the jaw and legs), breathing airflow (recording events of apnea or hypopnea), thoracic and abdominal breathing movements (distinguishing obstructive and central apnea), the oxygen saturation of blood (SpO_2_, monitoring the degree of hypoxia), electrocardiograph (ECG, detecting arrhythmia or heart abnormalities), posture sensor (recording the sleeping position and evaluating postural related respiratory events). Based on these parameters, we can analyze the patient’s sleep stages and their nighttime awakening situations, etc. To clarify the clinical efficacy of point-vagus nerve stimulation on adolescent insomnia, the analysis of sleep continuity indicators and sleep structure will be conducted, and the changes in brain electrical power spectrum will also be analyzed.

### 2.11 Trial assessment

This trial carries out compliance evaluation, analyzes the causes of dropouts, assesses adverse events, and conducts safety evaluations. These measures are designed to ensure the safety of the patients as well as the scientific validity and reliability of the trial results.

### 2.12 Changes to trial outcomes after trial commenced

We are committed to conducting this trial in strict accordance to the protocol. However, should any issues arise during the trial necessitating adjustments to certain factors, we will promptly apply for ethical review and notify all relevant parties. Any such adjustments will be disclosed in a transparent and detailed manner. We will exercise utmost with each modification to ensure the scientific rigor and reliability of the trial results.

### 2.13 Assignment of interventions

Single-blind study (Investigator, Outcomes Assessor): Individuals who evaluate the results will remain blind to the condition of the participants (intervention or control group). After the study participants were included in the TransHealth study, they received online access to the e-health platform. First, they were asked to complete questionnaires for baseline assessment (T0). Only after completing the T0 assessment, a 1:1 randomization for intervention and control group was carried out using a computer-based code with variable block length generated by the Institute for Medical Biometry and Epidemiology (Figures 1, 2). At the end of study participation, participants will complete the T1 assessment. Against this background, we do not expect any loss of data.

### 2.14 Data collection, management and analysis

The Case Report Form (CRF) will be used to collect study data, and EXCEL software will be employed for data collection or recording. Junying Du will be responsible for data management. At the end of the study, the investigator will submit the CRFs of all enrolled patients to the data management center, which should be complete and signed. A professional data management company will be commissioned for clinical data management. Data will be analyzed using the statistical software SPSS 19.0. Uniform testing SOPs will be developed by the subject group.

## 3 Discussion

The aim of this study is to assess the effectiveness of auricular-vagus nerve stimulation in adolescent insomnia patients and its impact on brain function regulation mechanisms. Through a randomized controlled trial, we expect to contribute scientific evidence for non-pharmacological insomnia treatment in adolescents. Adolescence is a crucial period in physical and mental development, where quality of sleep directly influences emotional and physical well-being (27). While short-term symptom reduction can be achieved with medication therapy like exogenous melatonin, its long-term use in adolescents is limited by potential adverse effects (28). Therefore, exploring safe and efficient non-pharmacological treatments is valuable. If the study confirms auricular point-vagus nerve stimulation as an effective treatment for teenage insomnia, it could offer a new therapeutic option, improving long-term prognosis and reducing reliance on medication. Investigating brain activity regulation mechanisms may reveal biological targets for more sophisticated intervention in insomnia management. However, the study has limitations. The findings may have limited generalizability due to the specific age range of the teenage sample. Additionally, the 8-week study duration might not provide a comprehensive assessment of long-term safety and efficacy. Variations in non-pharmacological interventions and individual responses could affect efficacy evaluation accuracy. Future research could address these limitations by increasing sample size, extending follow-up periods to evaluate long-term safety and effectiveness of auricular-vagus nerve stimulation, and investigating the impact of different stimulation levels and frequencies on treatment efficacy. Molecular biology and neuroimaging techniques could further elucidate the mechanism of action of auricular-vagus nerve stimulation.

### 3.1 Strengths and limitations

The study prevents a novel approach to addressing teenage insomnia by integrating modern neurophysiological strategies with traditional acupuncture. Utilizing a Randomized Controlled Trial design enhances the reliability of the results and minimizes bias. Objective measures, including polysomnography (PSG), HRV, and fMRI, are employed to comprehensively evaluate the treatment effects. By exploring the neural mechanisms underlying the treatment outcomes, the study aims to advance understanding of insomnia and its management. With a focus on non-pharmacological interventions due to concerns regarding medication side effects in adolescents, the generalizability of the findings may be limited to the teenage population. Conducted in a specific cultural context, the study’s findings may have restricted applicability beyond this setting. Despite efforts to recruit an adequate number of participants, the sample size limitations could affect the study’s ability to detect smaller effects. While the treatment and follow-up durations are clearly defined, the sustainability and long-term benefits of the intervention remain uncertain. Various factors contributing to insomnia may not have been fully considered in this study.

### 3.2 Ethics and dissemination

To ensure compliance with ethical standards and protect participants’ rights, the Ethics Committee must approve the project before implementation. Prior to commencing the study, participants and their legal guardians must provide informed consent by signing a formal document. All collected data will be treated confidentially, with information anonymized or coded to maintain privacy. Immediate action should be taken in response to any adverse events to uphold the accuracy and integrity of the gathered data. Researchers are required to disclose any potential conflicts of interest that may affect the study’s impartiality. For scientific rigor and validity, the research protocol, methodology, and results must be disseminated to the academic and broader communities through appropriate channels. The study outcomes should be published in peer-reviewed scientific journals. Patients and/or the public were involved in the design, or conduct, or reporting, or dissemination plans of this research. Refer to the Methods section for further details.

## Footnotes

^1^ http://www.chictr.org.cn

^2^ http://restfmri.net
